# Supplementary material for: Differentially Expressed microRNAs in MIA PaCa-2 and PANC-1 Pancreas Ductal Adenocarcinoma Cell Lines are Involved in Cancer Stem Cell Regulation
Source: Int J Mol Sci. 2019 Sep 10;20(18):4473. doi: 10.3390/ijms20184473 (PMC6770012; doi:10.3390/ijms20184473)
Supplement: Supplementary file 1 [file ijms-20-04473-s001.pdf]

Supplementary materials:

Table S1. The screened differentially expressed miRNAs between MIA PaCa-2 and PANC-1 cell lines.

MiRNAs	Name	Fold Change	*P* value
Down-regulated	miR-100-5p	0.016606436	0.000188637
	miR-31-5p	0.017226006	0.007538906
	miR-99a-5p	0.026843777	0.002133024
	miR-125b-5p	0.061107083	0.001063108
	miR-376a-3p	0.068207184	0.000418883
	miR-4324	0.078867382	0.000178371
	miR-125a-5p	0.080302228	0.002861962
	miR-376b-3p	0.083612342	0.001083977
	miR-495-3p	0.08585255	2.87489×10^{-5}
	miR-376c-3p	0.094054758	6.83902×10^{-5}
	miR-369-3p	0.107297103	9.6623×10^{-7}
	miR-196a-5p	0.127836439	0.000123521
	miR-9-5p	0.134515745	2.40619×10^{-5}
	miR-377-3p	0.152867063	2.00246×10^{-6}
	miR-196b-5p	0.163555363	7.7782×10^{-5}
	miR-487b	0.202678768	0.000353255
	miR-138-5p	0.206252959	1.14878×10^{-5}
	miR-127-3p	0.214520872	5.34956×10^{-5}
	miR-154-3p	0.233909146	0.000175925
	miR-424-5p	0.235831448	0.000147332
	miR-181b-5p	0.23655619	0.000816523
	miR-136-5p	0.245338461	0.002327353
	miR-329	0.262560844	5.81567×10^{-6}
	miR-337-5p	0.280325576	0.003346582
	miR-487a	0.283120119	0.001086959
	miR-181a-5p	0.290772767	0.001105691
	miR-181d	0.316232046	0.000741557
	miR-409-3p	0.317494342	0.00051799
Up-regulated	miR-221-3p	3.388619479	0.005989412
	let-7d-5p	4.126060815	0.00011837
	miR-7-5p	4.249069105	0.001781555
	miR-4468	4.667483306	0.000222253
	miR-135b-5p	5.315215062	0.001081975

Table S2. Sequences of RT primers, and PCR primers.

PT Primers	
**Name**	**Sequence (5'–3')**
miR-100-5p-RT	GTCGTATCCAGTGCAGGGTCCGAGGTATTCGCACTGGATACGACCACAAG
miR-31-5p-RT	GTCGTATCCAGTGCAGGGTCCGAGGTATTCGCACTGGATACGACAGCTAT
miR-99a-5p-RT	GTCGTATCCAGTGCAGGGTCCGAGGTATTCGCACTGGATACGACCACAAG
miR-376b-3p-RT	GTCGTATCCAGTGCAGGGTCCGAGGTATTCGCACTGGATACGACAACATG
miR-495-3p-RT	GTCGTATCCAGTGCAGGGTCCGAGGTATTCGCACTGGATACGACAAGAAG
miR-196a-5p-RT	GTCGTATCCAGTGCAGGGTCCGAGGTATTCGCACTGGATACGACCCCAAC
miR-9-5p-RT	GTCGTATCCAGTGCAGGGTCCGAGGTATTCGCACTGGATACGACTCATAC
miR-487b-3p-RT	GTCGTATCCAGTGCAGGGTCCGAGGTATTCGCACTGGATACGACAAGTGG
miR-138-5p-RT	GTCGTATCCAGTGCAGGGTCCGAGGTATTCGCACTGGATACGACCGGCCT
miR-127-3p-RT	GTCGTATCCAGTGCAGGGTCCGAGGTATTCGCACTGGATACGACAGCCAA
miR-154-3p-RT	GTCGTATCCAGTGCAGGGTCCGAGGTATTCGCACTGGATACGACAATAGG
miR-424-5p-RT	GTCGTATCCAGTGCAGGGTCCGAGGTATTCGCACTGGATACGACTTCAAA
miR-181b-5p-RT	GTCGTATCCAGTGCAGGGTCCGAGGTATTCGCACTGGATACGACACCCAC

miR-221-3p-RT	GTCGTATCCAGTGCAGGGTCCGAGGTATTCGCACTGGATACGACGAAACC
let-7d-5p-RT	GTCGTATCCAGTGCAGGGTCCGAGGTATTCGCACTGGATACGACAACTAT
miR-7-5p-RT	GTCGTATCCAGTGCAGGGTCCGAGGTATTCGCACTGGATACGACACAACA
miR-4468-RT	GTCGTATCCAGTGCAGGGTCCGAGGTATTCGCACTGGATACGACATCTCA
miR-135b-5p-RT	GTCGTATCCAGTGCAGGGTCCGAGGTATTCGCACTGGATACGACTCACAT
U6- RT	GGAACGCTTCACGAATTTG
**PCR Primers**	
**Name**	**Sequence (5'–3')**
miR-100-5p-FO	ACCCGTAGATCCGAA
miR-31-5p-FO	CTGCCGAGGCAAGATG
miR-99a-5p-FO	CGAACCCGTAGATCCG
miR-376b-3p-FO	CTGCCGATCATAGAGGAAAA
miR-495-3p-FO	TGCTGCCGAAACAAACATGG
miR-196a-5p-FO	GCTGCCGTAGGTAGTTTCA
miR-9-5p-FO	CTGCCGTCTTTGGTTATCT
miR-487b-3p-FO	TGCCGAATCGTACAGGGT
miR-138-5p-FO	GCCGAGCTGGTGTTGTG
miR-127-3p-FO	TGCCGTCGGATCCGTCT
miR-154-3p-FO	TGCTGCCGAATCATACACG
miR-424-5p-FO	GCCGCAGCAGCAATTCAT
miR-181b-5p-FO	CTGCCGAACATTCATTGCT
miR-221-3p-FO	CTGCCGAGCTACATTGTC
let-7d-5p-FO	TGCCGAGAGGTAGTAGGTT
miR-7-5p-FO	CTGCCGTGGAAGACTAGTG
miR-4468-FO	CTGCCGAGAGCAGAAG
miR-135b-5p-FO	GCCGTATGGCTTTTCATT
miR-RE	GTGCAGGGTCCGAGGT
U6-FO	ATTGGAACGATACAGAGAAGATT
U6-RE	GGAACGCTTCACGAATTTG

Table S3. Target genes of differentially expressed miRNAs.

miRNA	Targets
miR-100-5p	THAP2, SMARCA5, PPP1CB, MTOR, KBTBD8, HS3ST2, FKBP5, BMPR2
miR-31-5p	ZC3H12C, YWHAE, TBXA2R, SYDE2, STK40, SRC, SFXN1, SATB2, RHOBTB1, RDX, RASA1, RAB27A, PYURF, PRKCE, PPP2R2A, PHF12, NUMB, NFAT5, MMP16, LATS2, KLF13, JAZF1, HIF1AN, FZD3, FRK, EDC3, DPYSL5, DMD, CREG1, CDK1, CDC42SE1, CCNT1, BAHD1, ARID1A, AFF1
miR-99a-5p	TMEM30A, RAVER2, MTMR3, HOXA1, GRHL1, FGFR3
miR-376b-3p	ZNF780B, ZFHX3, ZFHX3, ZFHX3, WDR17, PPM1A, HNRNPA0, DCTN5, BEND4
miR-495-3p	ZNF703, ZBTB47, ZBTB37, ZBTB18, VGLL4, UHMK1, UBE2Z, SMOC1, SDC2, RNF138, RAB10, QSER1, PER2, PCDH1, MYO10, MTA3, MPRIP, MARCKS, KPNA2, KLHL15, KIAA1549L, KDM5B, HSPA5, HSP90AA1, HOXC8, DDIT4, CRLS1, CNBP, CDK1, CDCA2, CDC73, CD164, CBX4, BTF3L4, BMI1, ATP7A, ANKRD40, AGO2
miR-196a-5p	ZNF354B, ZCCHC3, YOD1, YIPF6, TSPAN12, TMEM194A, TGFBR3, SYT9, SPTSSA, SMCR8, SALL3, RGL2, RDX, RCC2, PRUNE2, PPP1R15B, POLR2D, PLEKHA3, NXPE3, NUP155, NRXN1, MAPK1, LRIG3, LCOR, KLHDC8B, KCTD21, KCNJ2, IGF2BP3, IGF2BP1, IGDCC4, HOXC8, HOXB8, HOXB7, HOXA9, HOXA7, HOXA5, HMGA2, HMGA1, HAND1, GRPEL2, GLTP, GATA6, FAM127B, FAM127A, FAM104A, EXOC8, EPHA7, E2F7, DYRK2, CDV3, CDKN1B, CCDC47, CALM1, C11orf57, BRAP, BACH1, ACER2
miR-9-5p	ZBED3, UHMK1, UBE4B, TRPM7, TGFBR2, TGFBI, TFRC, TESK2, TC2N, TBPL1, SYNGR2, SYAP1, STMN1, STK3, SRF, SPAG9, SOCS5, SNX7, SLC39A14, SLC35B3, SIRT1, SERINC5, SERAC1, SEC23IP, SDC1, RYBP, RNF44, RNF146, REST, RBFOX2, RAB34, PXDN, PTPRK, PRRX1, PRDM1, PPARA, POU2F2, POU2F1, PNRC2, PIGM, PI4K2A, PI4K2A, PDZK1, P4HA2, OPTN, ONECUT2, NR2E1, NFKB1, NFATC3, NCOR2, MYLK, MYH9, MTHFD2, MESDC1, MAP3K3, MAP1B, LDLRAP1, KLF5, KIF1C, KIAA1468, KCNJ2, IGF2R, ID4, HN1L, HIST1H4H, HIST1H2AI, HIST1H2AE, GPBP1L1, GIGYF1, FOXP1, FOXO3, FOXO1, FBN2, FAM73B, FAM46A, ETS1, EOGT, EN2, ELAVL1, EFNA1, EDEM3, DRD2, DICER1, CXCR4, CPEB4, COLEC12, CHSY1, CHMP2B, CERS2, CDX2, CDH1, CCNG1, CCNDBP1, CAPZA1, C9orf89, BCL6, BCL2L11, BACE1, AUH, ATP7A, AP3B1, ANP32B, AMOTL1

miR-487b	ZMYND8, SSBP3, PCF11, PAK7, NSMF, NRARP, KAT6B, ITGA6, GLCCI1, EFHD2
miR-138-5p	ZNF607, ZMYND11, VIM, UBE2V1, TP53INP2, TMEM189-UBE2V1, TMEM189, SOX4, SIRT1, SENP1, SEMA4C, ROCK2, RMND5A, RHOC, RELN, RARA, PTK2, PPM1L, PLAGL2, PHKG2, NR3C1, NEUROD1, MXD1, MEX3A, MAP3K11, LNPEP, LIN52, IGLON5, IGF2BP1, HMGA1, HIF1A, H2AFX, GRID1, GPR124, GNAI2, FERMT2, EZH2, EID1, DNAJB6, DEK, CCND3, CASP3, ARL5B, ARHGEF3, ARHGAP42, AGO1
miR-127-3p	SETD8, SEPT7, MAPK4
miR-154-3p	PRKAA1, ATP11C
miR-424-5p	ZNF704, ZNF449, ZNF367, ZFHX4, YTHDC1, WNK3, WIPI2, WEE1, USP42, UBR3, TRAK1, TMEM100, TLL1, TAOK1, SMAD7, SLC2A3, SETD1B, SEC24A, RARB, PTPRD, PRDM4, PPP1R11, PNRC2, MYO5A, MYB, MTMR3, MOB4, MAP2K1, LSM11, LRIG2, KIF23, HSPE1-MOB4, HNRNPA1, HCFC2, FOXK1, FKBP1A, EFNB2, DMTF1, DDX3X, CUL2, CRK, CREBL2, CDK17, CDK1, CDCA4, CDC37L1, CDC14A, CCNE1, CCND1, CAPZA2, C1orf21, BZW1, ARHGAP32, ANLN, AKT3
miR-181b-5p	ZNF268, ZFP69B, ZFP36L1, TMCC1, SLC25A37, RPS6KA3, PLAG1, OSBPL3, KIAA1551, GSKIP, FSD1L, DDX52, CBX7, C2orf69, BCL2
miR-221-3p	ZNF652, ZBTB37, UBE2N, TRPS1, TMEM132B, TMCC1, TIPARP, TIMP3, SRSF2, SPTSSA, SOX11, SOCS3, SOCS1, RNF44, RNF4, PTBP3, PPP6C, POGZ, PIK3R1, PHF12, PDIK1L, PDGFA, PCDHAC2, PCDHAC1, PCDHA8, PCDHA7, PCDHA6, PCDHA5, PCDHA4, PCDHA3, PCDHA2, PCDHA13, PCDHA12, PCDHA11, PCDHA10, PCDHA1, PAK1, OIP5, MYLIP, MIDN, MAPK10, LYSMD1, KIF16B, HNRNPA0, HMBOX1, HECTD2, FOS, DKK2, DDIT4, CTCF, CHSY1, CDKN1C, BRWD1, BCL2L11, BBC3, ATXN1, ARHGAP42, AGO2
let-7d-5p	ZNF774, ZNF644, ZNF566, ZNF280B, ZNF200, ZCCHC3, ZC3HAV1L, ZBTB39, YOD1, WASL, TXLNG, TSC22D2, TRIM71, TNFSF9, TMTC3, TMEM194A, TGFBR1, SYNJ2BP, SURF4, STK40, SREK1IP1, SREK1, SMCR8, SLC30A6, SLC16A9, SENP5, SEMA4C, SALL3, RRM2, RNFT1, RDX, POLR3D, PMAIP1, PLXND1, PLEKHO1, PLEKHA3, PGRMC1, PGM2L1, PEX11B, PEG10, PDZD8, PDE12, PCGF3, ONECUT2, NSD1, NHLRC2, NCOA1, NAA30, NAA20, MXD1, MSI2, MLLT10, MDM4, MBD2, LRIG3, LIN28B, LBR, KMT2D, KLHDC8B, KIAA0930, KIAA0391, KATNAL1, IPO9, IGF2BP3, IGF2BP1, IGDCC4, HMGA2, HAND1, GOLT1B, GOLGA4, FIGN, FAM43A, FAM222B, FAM104A, EPHA4, EIF4G2, EFHD2, EDN1, EDEM3, E2F6, DVL3, DUSP1, DNA2, DLC1, DICER1, CPA4, CLDN12, CELF1, CDV3, CDKN1A, CCNT2, CCND1, CBX5, CALU, C5orf51, BACH1, ATXN7L3B, ATXN7L3, ARID3B, ARID3A, ANKRD46, ADIPOR2, ADCY9
miR-7-5p	ZNF805, ZFAND4, ZC3H4, ZBTB22, YOD1, XIAP, VPS4A, VPS26A, UBXN2B, UBQLN4, TRMT13, TNRC6B, TNRC6A, TIMM50, TFRC, TAF1, STK11, SRSF1, SRGAP2, SQSTM1, SPTY2D1, SPATA2, SOCS2, SNCA, SMARCD1, SLC6A9, SLC5A3, SLC5A3, SLC35A5, SLC25A37, SLC25A15, SETD8, SERTAD3, SERP1, SEMA6D, RYK, RSPRY1, RRAS2, RCC2, RBMS3, RAF1, RAD54L2, RAB11FIP5, PURB, PTAR1, PSME3, PPIF, POLE4, POGK, PLEC, PIK3R3, PIK3CD, PIK3CB, PIGH, PFN2, PDE4D, PAX6, PARP1, PAPPA, OSBPL11, NXT2, NUDCD3, NREP, NR1H2, NIPAL3, NDUFA4, NDFIP2, MAP1B, LUC7L2, LSM12, LRRC59, KPNA6, KLF4, KIF16B, KIAA0247, IRS2, IRS1, IGSF8, IGF1R, IDE, HECTD3, HDLBP, HAP1, GJC1, GATAD2B, GATA6, GALNT3, FXR1, FNDC4, FAM126B, EXOSC2, EIF4EBP2, EIF4E, EHD1, EGFR, DNAJC5, CUL5, CRLS1, CNOT8, CNN3, CKAP4, CHAMP1, CCNT2, CAPZA1, CAMK2D, CALU, CACNG7, C5orf22, C20orf24, C1orf21, BMPR2, ATXN1, ASXL1, ARF4, ALG9, AKT3, ADCY9, ABCC1
miR-4468	ZNF843, ZNF585B, ZNF107, ZNF106 , YWHAE, YARS, XKR4, WSB1, WBP2, UBXN2A, TSTD2, TRIM71, TNRC6A, TNPO1, TMEM255B, TM4SF1, TFRC, TFCP2L1, TEAD3, TBC1D13, STX7, ST3GAL5, SMS, SH2D4A, SCD, RPS28, RPL37A, RNF185, REXO1, RBPJ, RBM3, RACGAP1, RAB2A, RAB23, PTP4A1, PRICKLE2, PPIL1, POC1A, PLEKHA6, PLAGL2, PKP1, PER1, PARVG, PAQR7, NRAS, NPM1, NDUFA10, MYPN, MSANTD4, MFSD9, MCUR1, MAPRE1, LUC7L3, LRWD1, LRRC4B, LEPRE1, KRR1, KLHL15, KIF3B, ITGA2, IBA57, HOXA3, HAUS3, HAND2, GOLIM4, GOLGA8B, GOLGA8A, GATAD2B, FZD6, FMNL3, FCF1, FBXL20, FAM46A, FAM127A, EMB, EFNA3, EFNA3, E2F6, DYRK3, DOCK11, DNAH9, DHX33, DGAT1, DCTN5, CNIH4, CHMP4B, CENPM, CDK1, CAV1, CAMSAP1, CALR, C14orf2, C11orf84, BRPF1, ATXN1, ATP8B3, ATG14, ASGR2, APBB3, ALG14, ALDOA, ALDH3B1, AK2
miR-135b-5p	TGFBR1, SMAD5, SMAD5, NUFIP2, LZTS1, LRRC15, KLF4, ARC, APC, ACVR1B

Table S4. Enriched Gene Ontology (GO) terms in molecular function (MF), biological process (BP) and cellular component (CC) categories for target genes of the 18 differentially expressed miRNAs.

GO_ID	GO_term	Category	Count	Target genes	FDR

GO:0005634	nucleus	CC	303	ZFHX3, XIAP, WASL, TIMP3, STK3, STK11, SRGAP2, SRF, SRC, SOCS1, SMAD5, SIRT1, RPS6KA3, ROCK2, REST, RARB, RARA, RAF1, RAB2A, PTK2, PRKCE, PRKAA1, PRDM4, PPP1CB, PPM1A, PPARA, POLE4, PIK3R1, PIK3CB, PCGF3, PAX6, NR1H2, NFKB1, NFATC3, MYB, MTOR, MDM4, MAPK1, MAP2K1, KIF23, ITGA2, IRS1, IGF2BP1, ID4, HSP90AA1, HMGA2, HIF1A, HAND1, FOXO3, FOXO1, FOS, FGFR3, EZH2, ETS1, ELAVL1, EGFR, DUSP1, DICER1, DDX3X, CRK, CPEB4, CDKN1B, CDKN1A, CDK1, CCNE1, CCND3, CCND1, CASP3, CAMK2D, CALR, CALM1, BMI1, BCL6, BCL2, APC	1.67360×10^{-17}
GO:0005515	protein binding	MF	440	ZFHX3, YWHAE, XIAP, WASL, VIM, TIMP3, TGFBR2, TGFBR1, TFRC, TBPL1, TAOK1, STMN1, STK3, STK11, SRGAP2, SRF, SRC, SOCS5, SOCS3, SOCS2, SOCS1, SMAD5, SIRT1, SEMA4C, SDC2, SDC1, RRAS2, RPS6KA3, ROCK2, RGL2, REST, RDX, RASA1, RARA, RAF1, RAB2A, RAB10, PTK2, PRKCE, PRKAA1, PRDM4, PPP2R2A, PPP1CB, PPM1A, PPIF, PPARA, POLE4, PIK3R3, PIK3R1, PIK3CD, PIK3CB, PHKG2, PFN2, PDGFA, PCGF3, PAX6, PARVG, PAK1, NR1H2, NFKB1, NFATC3, MYO10, MYLK, MYB, MTOR, MDM4, MAPK10, MAPK1, MAP3K3, MAP3K11, MAP2K1, KLF4, KIF23, ITGA6, ITGA2, IRS2, IRS1, IGF2BP1, IGF1R, ID4, HSP90AA1, HMGA2, HIF1A, HAND1, GNAI2, FZD6, FZD3, FOXO3, FOXO1, FOS, FKBP5, FGFR3, EZH2, ETS1, EPHA7, EPHA4, ELAVL1, EIF4E, EGFR, EFNB2, EFNA3, EFNA1, EDN1, DVL3, DUSP1, DRD2, DPYSL5, DICER1, DDX3X, CXCR4, CUL2, CRK, CPEB4, CERS2, CDKN1B, CDKN1A, CDK1, CDH1, CCNG1, CCNE1, CCND3, CCND1, CAV1, CASP3, CAMK2D, CALR, CALM1, BRAP, BMPR2, BMI1, BCL6, BCL2L11, BCL2, APC, AKT3, ACVR1B	7.33032×10^{-17}
GO:0005654	nucleoplasm	CC	185	ZFHX3, XIAP, STK11, SRGAP2, SRF, SMAD5, SIRT1, RPS6KA3, REST, RARB, RARA, PRKAA1, PPP2R2A, PPP1CB, PPM1A, PPARA, PAX6, NR1H2, NFKB1, NFATC3, MTOR, MDM4, MAPK10, MAPK1, KLF4, KIF23, HSP90AA1 , HMGA2, HIF1A, HAND1, GNAI2, FOXO3, FOXO1, FOS, FKBP5, EZH2, ETS1, ELAVL1, CUL2, CDKN1B, CDKN1A, CDK1, CCNG1, CCNE1, CCND3, CCND1, CASP3, CAMK2D, CALM1, BMI1, BCL6, APC, AKT3	2.41211×10^{-15}
GO:0000122	negative regulation of transcription from RNA polymerase II promoter	BP	74	ZFHX3, SIRT1, REST, RARB, RARA, PPM1A, PPARA, PAX6, NR1H2, NFKB1, MYB, MDM4, KLF4, ID4, HMGA2, HAND1, FOXO3, FOXO1, EZH2, EFNA1, EDN1, DICER1, CCND1, CAV1, CALR, BMI1, BCL6	5.44009×10^{-12}
GO:0045944	positive regulation of transcription	BP	84	SRF, SIRT1, RPS6KA3, REST, RARB, RARA, RAF1, PPARA, PIK3R1, PAX6, NR1H2, NFKB1, NFATC3, MYB, KLF4, ITGA6, ID4,	1.59992×10^{-9}

	from RNA polymerase II promoter			HMGA2, HIF1A, HAND1, FOXO3, FOXO1, FOS, ETS1, EGFR, EDN1, DRD2, DDX3X, BMPR2, ACVR1B	
GO:0045893	positive regulation of transcription, DNA-templated	BP	53	ZFHX3, TGFBR1, SRC, SMAD5, REST, RARA, PPM1A, PPARA, PAX6, NR1H2, NFKB1, NFATC3, MYB, MAPK1, MAP2K1, KLF4, HMGA2, HIF1A, FOXO3, FOXO1, FOS, ETS1, DVL3, CDH1, CCNE1	8.17533 × 10^{-8}
GO:0005737	cytoplasm	CC	266	ZFHX3, XIAP, VIM, TBPL1, TAOK1, STMN1, STK3, STK11, SRGAP2, SRF, SRC, SOCS5, SOCS3, SOCS2, SOCS1, SMAD5, SIRT1, SEMA6D, SDC1, RPS6KA3, RELN, RDX, RASA1, RARB, RARA, RAF1, PTK2, PRKCE, PRKAA1, PRDM4, PIK3R1, PFN2, PAX6, PARVG, PAK1, NR1H2, NFKB1, NFATC3, MYO10, MYLK, MTOR, MAPK10, MAPK1, MAP3K3, MAP3K11, MAP2K1, KLF4, IRS1, IGF2BP1, ID4, HSP90AA1, HIF1A, HAND1, GNAI2, FZD3, FOXO3, FOXO1, EZH2, ETS1, EPHA4, ELAVL1, EIF4E, EGFR, EDN1, DUSP1, DPYSL5, DICER1, DDX3X, DDIT4, CXCR4, CRK, CPEB4, CDKN1B, CDK1, CDH1, CCND3, CCND1, CASP3, CAMK2D, CALR, CALM1, BRAP, BMPR2, BMI1, BCL2, APC, AKT3	1.0343 × 10^{-8}
GO:0005829	cytosol	CC	181	YWHAE, XIAP, WASL, VIM, TGFBR2, TAOK1, STMN1, STK3, STK11, SRGAP2, SRC, SOCS3, SOCS2, SOCS1, SMAD5, RPS6KA3, ROCK2, REST, RASA1, RAF1, PTK2, PRKCE, PRKAA1, PPP2R2A, PPP1CB, PPM1A, PIK3R3, PIK3R1, PIK3CD, PIK3CB, PHKG2, PAK1, NFKB1, NFATC3, MYO10, MYLK, MTOR, MAPK10, MAPK1, MAP3K3, MAP2K1, KIF23, IRS2, IRS1, IGF2BP1, HSP90AA1, HIF1A, GNAI2, FOXO3, FOXO1, FOS, ELAVL1, EIF4E, DVL3, DPYSL5, DICER1, DDIT4, CUL2, CRK, CDKN1B, CDKN1A, CDK1, CCNE1, CCND1, CASP3, CAMK2D, CALR, CALM1, BRAP, BCL2L11, BCL2, APC	9.65795 × 10^{-7}
GO:0030335	positive regulation of cell migration	BP	27	TGFBR1, SEMA6D, SEMA4C, RRAS2, RDX, PTK2, PIK3R1, PIK3CD, PDGFA, PAK1, MYLK, MAPK1, ITGA6, IRS2, IGF1R, EGFR, EDN1, APC	7.55645 × 10^{-6}
GO:0008134	transcription factor binding	MF	35	SRF, SIRT1, REST, RARA, PPARA, PIK3R1, PAX6, NFKB1, MAPK1, HMGA2, HIF1A, HAND1, FOS, ETS1, DDX3X, CCND1, BCL2	3.38754 × 10^{-6}

FDR: false discovery rate.

Table S5. The top ten enriched pathways for target genes of the 18 differentially expressed miRNAs.

Pathway_ID	Name	Count	Target Genes	FDR
hsa05206	MicroRNAs in cancer	41	VIM, TRIM71, TIMP3, STMN1, SOCS1, SIRT1, RDX, RAF1, PRKCE, PDGFA, NFKB1, MTOR, MDM4, MARCKS, MAP2K1, KIF23, IRS2, IRS1, IGF2BP1, HMGA2, FZD3, FGFR3, EZH2, EGFR, DICER1, DDIT4, CRK, CDKN1B, CDKN1A, CCNG1, CCNE1, CCND1, CASP3, BMPR2, BMI1, BCL2L11, BCL2, APC	2.39217 × 10^{-9}

hsa04068	FoxO signaling pathway	26	TGFBR2, TGFBR1, STK11, SIRT1, RAF1, PRKAA1, PIK3R3, PIK3R1, PIK3CD, PIK3CB, MAPK10, MAPK1, MAP2K1, IRS2, IRS1, IGF1R, FOXO3, FOXO1, EGFR, CDKN1B, CDKN1A, CCND1, BCL6, BCL2L11, AKT3	9.87930×10^{-8}
hsa05215	Prostate cancer	21	RAF1, PIK3R3, PIK3R1, PIK3CD, PIK3CB, PDGFA, NFKB1, MTOR, MAPK1, MAP2K1, IGF1R, HSP90AA1, FOXO1, EGFR, CDKN1B, CDKN1A, CCNE1, CCND1, BCL2, AKT3	2.08253×10^{-7}
hsa04917	Prolactin signaling pathway	19	SRC, SOCS5, SOCS3, SOCS2, SOCS1, RAF1, PIK3R3, PIK3R1, PIK3CD, PIK3CB, NFKB1, MAPK10, MAPK1, MAP2K1, FOXO3, FOS, CCND1, AKT3	2.55548×10^{-7}
hsa04550	Signaling pathways regulating pluripotency of stem cells	26	ZFHX3, SMAD5, REST, RAF1, PIK3R3, PIK3R1, PIK3CD, PIK3CB, PCGF3, PAX6, MAPK1, MAP2K1, KLF4, IGF1R, ID4, HAND1, FZD6, FZD3, FGFR3, DVL3, BMPR2, BMI1, APC, AKT3, ACVR1B	2.64961×10^{-7}
hsa05205	Proteoglycans in cancer	31	TIMP3, SRC, SDC2, SDC1, RRAS2, ROCK2, RDX, RAF1, PTK2, PPP1CB, PIK3R3, PIK3R1, PIK3CD, PIK3CB, PAK1, MTOR, MAPK1, MAP2K1, ITGA2, IGF1R, HIF1A, FZD6, FZD3, EGFR, CDKN1A, CCND1, CAV1, CASP3, CAMK2D, AKT3	3.41708×10^{-7}
hsa05200	Pathways in cancer	45	XIAP, TGFBR2, TGFBR1, ROCK2, RARB, RARA, RAF1, PTK2, PIK3R3, PIK3R1, PIK3CD, PIK3CB, PDGFA, NFKB1, MTOR, MAPK10, MAPK1, MAP2K1, ITGA6, ITGA2, IGF1R, HSP90AA1, HIF1A, GNAI2, FZD6, FZD3, FOXO1, FOS, FGFR3, EGFR, DVL3, CXCR4, CUL2, CRK, CDKN1B, CDKN1A, CDH1, CCNE1, CCND1, CASP3, BCL2, APC, AKT3	3.95094×10^{-7}
hsa05214	Glioma	17	RAF1, PIK3R3, PIK3R1, PIK3CD, PIK3CB, PDGFA, MTOR, MAPK1, MAP2K1, IGF1R, EGFR, CDKN1A, CCND1, CAMK2D, CALM1, AKT3	4.28379×10^{-6}
hsa04012	ErbB signaling pathway	19	SRC, RAF1, PTK2, PIK3R3, PIK3R1, PIK3CD, PIK3CB, PAK1, MTOR, MAPK10, MAPK1, MAP2K1, EGFR, CRK, CDKN1B, CDKN1A, CAMK2D, AKT3	8.83122×10^{-6}
hsa04066	HIF-1 signaling pathway	20	TFRC, PIK3R3, PIK3R1, PIK3CD, PIK3CB, NFKB1, MTOR, MAPK1, MAP2K1, IGF1R, HIF1A, EIF4E, EGFR, EDN1, CUL2, CDKN1B, CDKN1A, CAMK2D, BCL2, AKT3	1.04333×10^{-5}
